# Changes in consumption of added sugars from age 13 to 30 years: a systematic review and meta‐analysis of longitudinal studies

**DOI:** 10.1111/obr.12588

**Published:** 2017-09-04

**Authors:** E. M. Winpenny, T. L. Penney, K. Corder, M. White, E. M. F. van Sluijs

**Affiliations:** ^1^ MRC Epidemiology Unit and Centre for Diet and Activity Research (CEDAR) University of Cambridge Cambridge UK

**Keywords:** Adolescent, diet, longitudinal, sugar

## Abstract

Added sugar intake during adolescence has been associated with weight gain and cardiometabolic risk factors. Moreover, dietary habits may persist into adulthood, increasing chronic disease risk in later life. This systematic review investigated changes in intake of added sugars between the ages of 13 and 30 years.

Literature databases were searched for longitudinal studies of diet during adolescence or early adulthood. Retrieved articles were screened for studies including multiple measures of intake of sugars or sugary foods from cohort participants between the ages of 13 and 30. Data were analysed using random‐effects meta‐analysis, by the three main nutrient and food group categories identified (PROSPERO: CRD42015030126).

Twenty‐four papers reported longitudinal data on intake of added sugar or sucrose (n = 6), sugar‐sweetened beverages (SSBs) (n = 20) and/or confectionery (n = 9). Meta‐analysis showed a non‐significant per year of age decrease in added sugar or sucrose intake (−0.15% total energy intake (95%CI −0.41; 0.12)), a decrease in confectionery consumption (−0.20 servings/week (95%CI −0.41; −0.001)) and a non‐significant decrease in SSB consumption (−0.15 servings/week (95%CI −0.32; 0.02)). Taken together, the overall decrease in added sugar intake observed from adolescence to early adulthood may suggest opportunities for intervention to further improve dietary choices within this age range.

## Introduction

Sugar intake in childhood and adolescence, specifically intake of sugar‐sweetened beverages (SSBs) has been associated with weight gain in prospective cohort studies and randomized controlled trials 1, while substitution of SSBs with other beverages has been associated with reduced body fatness 2. Furthermore, increases in SSB consumption during adolescence have been associated with increases in body mass index (BMI) as well as overall cardiometabolic risk 3. There is concern that poor dietary habits, such as high sugar intake, developed in childhood persist into adulthood 4, increasing risk of obesity, metabolic syndrome and type 2 diabetes in later life 1, 5, 6, 7, 8.

Intake of added sugars is of particular concern for health 9, with the World Health Organization recommending that intake of free sugars (added sugars together with sugars naturally present in fruit juices, honey and syrups) should be less than 10% of total energy intake 10. Nevertheless, intake is high among younger age groups across many countries 11, 12, 13, 14, with added sugars comprising 12.8% of total energy intake among Australian adolescents 12 and 17.3% of total energy intake among US adolescents 14. The greatest contributors to high intake of added sugars in this age group, by food source, were sugary drinks and confectionery 14, 15.

The period of late adolescence to early adulthood has been suggested as an important, but overlooked age for establishment of long‐term health behaviour patterns 16. This is a period of transition 17, 18, which may allow disruption of an individuals' pre‐existing habits 19, 20 and therefore present opportunities for intervention to improve diet and long term health. In order to develop and target public health interventions most appropriately, it is important to understand underlying patterns in dietary behaviour throughout this period, the establishment of long‐term behaviour patterns and factors that impact on changes in behaviour. Studies have suggested that intake of free sugars may increase during childhood 21 and intake of sugary beverages may increase during adolescence 22. However, there has to date been no synthesis of evidence on change in intake of added sugars through adolescence and into early adulthood. In this review, we focus on longitudinal observational studies reporting intake of sugar, and sugary foods and drinks, in order to answer the question: How does intake of added sugars change from age 13 to age 30 years, and what are the determinants of such changes?

## Methods

### Literature search

Our literature search was conducted as part of a broader literature search of longitudinal observational studies that provided data on diet and physical activity in adolescence and early adulthood, reported in a scoping review of this topic 23. This search looked for prospective longitudinal data on dietary intake reported between mean ages of 13 and 30 years. We begin our review at age 13 years, because we are interested in transitions from mid‐adolescence into early adulthood. While a consensus has not been reached on a definition of early adulthood, Rindfuss (1991) suggested the 30th birthday as the end of the young adult years 18. This age range includes that of ‘emerging adulthood’ which has been postulated as a transitionary period between adolescence and full adulthood from age 18 to 25 years 24.

We searched seven electronic databases (Medline via PubMed, SCOPUS, Embase via Ovid, PsycInfo via EBSCO, ASSIA via ProQuest, Sportdiscus, and Web of Science Core Collection) from 1980 until January 2016. Search terms combined diet and physical activity outcomes, longitudinal methods and indicators of adolescent or young adult age range. We only searched for papers published after 1980 since data collection post‐1980 was one of our inclusion criteria to maximize relevance of findings for current and future populations. The full search strategy as applied to Medline is shown in the supporting information (Table S1). We also conducted additional hand searches; checking the reference lists of all included papers, and citation searches of key included papers, which addressed the same broad research question as our scoping review 23. This review has been registered on PROSPERO (PROSPERO ref: CRD42015030126).

### Study selection

The papers identified from the literature search were first screened and analysed as part of a scoping review of longitudinal data from adolescence to early adulthood across all macronutrient and food group diet outcomes 23. This scoping review demonstrated sufficient availability of data on sugary foods and drinks to warrant further systematic review on the overall topic of change in added sugar intake in this age range. All papers included in this scoping review (*n* = 98) were re‐assessed against the inclusion and exclusion criteria for the current review (Table 1) by two researchers (EW and TP) independently. Inclusion in this review was limited to those papers that reported longitudinal prospective data on consumption of sugar (as a nutrient) or sugary foods or drinks (SSBs), including at least two measurements at least one year apart between the ages of 13 and 30 years. Our definition of sugary foods was broad, encompassing any foods likely to have a high added sugar content, such as confectionery, cakes, biscuits, puddings. SSBs included drinks containing added sugars, including soft drinks, fruit flavoured or fruit‐based drinks and high‐energy drinks, sweetened milk drinks and sweetened teas and coffees, but excluding pure fruit juices and ‘diet’ drinks (zero or very‐low‐calorie drinks with artificial sweeteners). Studies could be situated in any country, but inclusion was restricted to papers published in English.

**Table 1:** Inclusion and exclusion criteria for the review

	Inclusion criteria	Exclusion criteria
Setting	Any country Studies where specified data (see below) were collected during or after 1980	Studies reporting on data collected prior to 1980
Participants	Those aged between 13 and 30 years, inclusive	Those aged below 13 years of age or above 30 years of age Participant groups selected based on a pre‐existing health condition (including obesity, eating disorders, malnutrition) or pregnancy Studies where participants are only competitive athletes
Outcomes	Any measure of sugar intake, including: Intake of total sugar, added sugars or sucrose. Intake of sugary foods (confectionery, cakes, biscuits, puddings) or drinks (soft drinks (excluding diet drinks), fruit drinks (other than pure juice), sugar‐sweetened beverages)	Studies including no measure of sugar intake Studies reporting tracking of sugar intake only with no data on absolute change in behaviour Studies reporting on diet quality indices or dietary patterns only
Study type	Longitudinal prospective quantitative studies, with data reported including on specified outcomes at atleast 2 time points (minimum 1 year apart) where mean age of the cohort is between ages 13 and 30 inclusive	Cross‐sectional studies Intervention studies or trial analyses Case–control studies Retrospective studies Qualitative studies Reviews
Publication type	Journal article	Conference abstract, study protocol, report, dissertation, book and professional journal
Language	English	All other languages

### Data extraction

Data were extracted from each included paper by one researcher (EW) and checked by a second researcher (TP). Any discrepancies were resolved by discussion between the two researchers. Data extracted from each paper included study (cohort) name, country of cohort, ethnicity, sex and socio‐economic status of participants, and the year at which participants would have been age 13, where reported. Further details were extracted for each wave of data collection within the age range of interest: sample size, mean age of the cohort, mean and standard deviation (SD) (or median and interquartile range where mean and SD were not available) of intake of sugar or sugary food or drinks (with units), and method of diet data collection. Depending on the outcomes reported, we also extracted any additional detail on the types of foods or drinks that were included in the reported data. Where studies addressed the determinants of changes in added sugar intake, we extracted information on reported associations between determinants and change in intake.

### Data preparation

Data on sugar and sugary food or drink outcomes were reported according to three distinct outcomes, which we chose to analyse separately: (i) added sugar or sucrose intake; (ii) SSB intake and (iii) confectionery intake. Data on added sugar and sucrose were reported together as these provided nutrient level measures of dietary sugar intake. SSBs and confectionery were the two main food groups on which data were reported in the studies reviewed. We defined SSBs as any sweetened beverages not presented as diet or non‐caloric beverages. For each outcome, where papers reported data across the same age range, one paper representing each study was selected for use in further quantitative analysis. Papers selected were those with the highest number of waves of data reported, those in which we were able to convert the reported data to the common metric for each diet outcome and the largest sample size. Data extracted from the selected papers were converted to a common metric for each diet outcome, to enable comparison between studies. The metrics chosen were percentage of total energy intake for sucrose and added sugar intake, and servings per week for food and drink intakes; these were the most common metrics reported. Data were converted to the appropriate units, and manipulated where necessary to give an overall mean and SD for the whole cohort for each food group at each wave. Further details on the calculations performed on data from each study are documented in the Supporting Information (Table S2). In order to examine the effect of calendar time on changes in sugar intake, we used data from each study indicating when the cohort was mean age 13 to calculate the year at each wave of data collection.

### Quality assessment

Included papers were assessed for methodological quality using a 10‐item quality assessment scale (see Table S3), based on that used previously 25, 26, 27, 28, which is specifically designed for longitudinal observational studies of diet. Papers from the same study were assessed separately because they may contribute independently to the analysis of different outcomes. The scale assessed four dimensions of methodological quality: (i) study population and participation rate; (ii) study attrition; (iii) data collection and (iv) data analysis. Two reviewers (EW and TP) independently assessed each paper, with discussion to resolve any disagreements. The total score was reported for each paper to provide an indication of the overall quality of the paper.

### Data analysis

Data on added sugar intake were analysed across the three main outcome categories: sucrose or added sugar, SSBs and confectionery. Quantitative data analysis was conducted using STATA (version 14). For each study, we regressed mean intake on average age of the cohort, weighted by number of participants at each data collection, to give change in intake per year across each study. From these initial analyses, the regression coefficient became the effect size estimate to be used in meta‐analysis, with the standard error (SE) of the beta coefficient used for weighting the meta‐analysis. For those studies with only two data points, where SEs were not available from the regression, SEs were calculated from the sample SDs. Random effects meta‐analysis was used, to account for differences between studies, using the method of DerSimonian and Laird, with the estimate of heterogeneity being taken from the inverse‐variance fixed‐effect model. The heterogeneity of associations was expressed using the I^2^ statistic. Where heterogeneity was high (I^2^ > 50%) and the analysis included more than 10 studies 29 (SSB meta‐analysis), we conducted meta‐regression and sub‐group analyses to explore causes of heterogeneity. Meta‐regression was used to test for potential effect modifiers: method of diet data collection, overall study quality score, overall time period of the study (using the middle calendar year of the data included) and overall age range of the study (using the middle age of the data included). We conducted sub‐group analyses by gender (where data were reported separately), by definition of SSB (1, beverages clearly specified as sugar‐sweetened vs. 2, beverages defined more generally, as soft drinks or fruit drinks, but not specified as sugar‐sweetened or diet) and by age (13 to 18 vs. 18 to 30 years). Subgroup analysis by age included only data points that were in the appropriate age category and studies that had at least two data points within the appropriate age category. Thus, for some studies, only a subset of data points were included and studies that did not include at least two data points within the age category were excluded. For the added sugar and sucrose meta‐analysis, we conducted stratified meta‐analyses *post‐hoc* treating added sugar and sucrose as two separate outcomes, rather than together in a combined meta‐analysis, to explore differences between these two measures.

To identify publication bias and small‐study effects we used funnel plot asymmetry for each outcome, as assessed visually and using Egger's test of bias 30, where more than 10 studies were included in meta‐analyses. As recommended, tests of funnel plot asymmetry were not used for meta‐analysis across fewer than 10 studies 29.

For studies that could not be included in the meta‐analysis, due to inability to convert data into standardized metrics or lack of detail on population variance, we present a brief narrative summary of study findings. Data on determinants of changes in added sugar intake are presented as a narrative synthesis across all added sugar outcomes.

Results are reported in accordance with the PRISMA statement 31.

## Results

**Figure 1:**
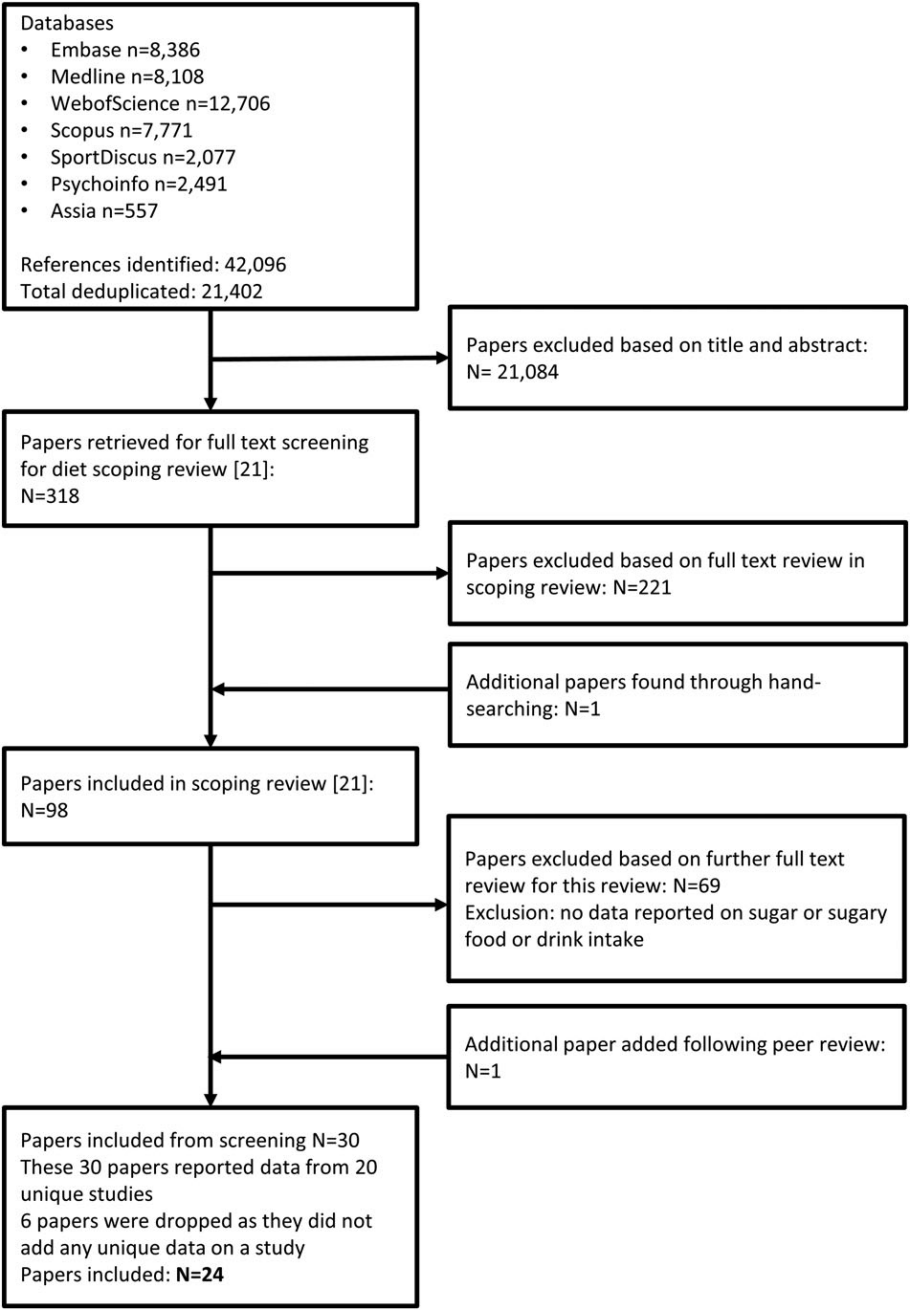
Flow chart of literature search and paper selection process.

Our initial searches identified a total of 21,402 records following the removal of duplicates. After initial screening of titles and abstracts, we considered 319 references for full text review for the published scoping review, which included 98 papers 23. Further screening of full texts identified 29 papers that reported longitudinal data on intake of sugar or sugary foods and drinks between the ages of 13 and 30 that were eligible for inclusion in this review (Fig. 1). One paper that was not identified in our database searches was added following peer review. Six papers were excluded following data extraction, because they did not provide unique data from a study, leaving a total of 24 included papers. Within these 24 papers, six papers reported on added sugar or sucrose intake, 20 papers reported on SSB consumption, and nine papers reported on confectionary consumption. Thirteen papers reported on multiple outcomes. Three papers also reported on consumption of cakes and biscuits 32, buns and biscuits 33 and pastries 34. However, these are not included in the quantitative analyses due to the small number of studies and diversity of food types included.

Table 2 presents an overview of the included studies, setting out how the individual papers contributed data to the quantitative outcomes and analysis of determinants. Table 2 also presents the quality assessment score for each study. Initial duplicate assessment of paper quality showed agreement on total score between reviewers (EW, TP) on 19 of the 24 papers (79%), with remaining differences in ratings resolved through discussion. Paper quality assessment scores ranged from 5 to 8 out of a maximum score of 10. Fifty‐eight percent of papers achieved a quality score of 7 or above. The lowest scoring item across the papers was ‘Presentation of data showing non‐selective non‐response during follow‐up measurement(s)’ which received a positive score for only one of the 24 papers (4%). In addition few papers (*n* = 3; 13%) scored positively on ‘Adjustment for misreporting’. The majority of papers scored positively on ‘Adequate description of sampling frame, recruitment methods, period of recruitment and place of recruitment’ (*n* = 23; 96%) and ‘Provision of exact information on follow‐up duration’ (*n* = 24; 100%). Table 2 additionally reports on the method of diet data collection because this is a major contributor to data quality 35. We distinguish between food frequency questionnaires (FFQs), which seek to achieve a comprehensive assessment of dietary intake over a given period, and ‘Questionnaires’ where a smaller number of targeted questions have been asked about particular food items.

**Table 2:** Overview of included papers, with details on their inclusion in reporting on sucrose, SSBs, confectionery and determinants of change in consumption, and quality assessment score

Reference	Study name	Country where study situated	Sample size and cohort characteristics*	Mean ages included (years)	Year at mean age 13	Method of diet data collection	Sucrose or added sugar	SSBs	Confectio‐nery	Determi‐nants	Quality assessment score
Adair LS *et al.* (2005) 36	Cebu Longitudinal Health and Nutrition Study (CLHBS)	Philippines	n range from 2040 to 2106	15, 19	1996	Two 24‐h dietary recalls	X	G	X	X	6
Ambrosini GL *et al.* (2013) 3	Western Australian Pregnancy (Raine) cohort	Australia	n range from 1009 to 1667	14, 17	2002–2005	FFQ	X	N/P	X	X	8
Astrom, A. N. *et al.* (2004) 49	The Norwegian Longitudinal Health Behaviour (NLHB) Study	Norway	n range from 627 to 963	15, 18, 19, 21, 23	1990	Questionnaire	X	D	✔	X	7
Bruno‐Ambrosius, K. *et al.* (2005) 47	—	Sweden	*n* = 162, all female	13, 14, 15	2000	Questionnaire	X	N/P	N/P	X	5
Davis, J. N. *et al.* (2009) 43	Study of Latino Adolescents at Risk for Diabetes (SOLAR) cohort	US	*n* = 85, all Latino	14, 15	2002	Two 24‐h dietary recalls	✔	✔	X	X	6
Deheeger, M. *et al.* (2002) 63	The French longitudinal study of growth and nutrition (FLSGN)	France	*n* = 94	14, 16	1998 (estimated)	Diet history method interview	✔	X	X	X	7
Falbe, J. *et al.* (2014) 37	Growing Up Today Study II (GUTS2)	US	*n* = 8,272	15, 17	2004	FFQ	X	✔	✔	✔	8
Feeley, A. *et al.* (2012) 48	Birth to Twenty (Bt20) study	South Africa	*n* = 1,451	13, 15, 17	2003	Questionnaire	X	✔	✔	X	6
Fiorito, L. M. *et al.* (2010) 64	—	US	*n* = 166, all female, non‐Hispanic white	13, 15	2004	Three 24‐h dietary recalls	✔	✔	X	X	6
Kvaavik, E. *et al.* (2005) 45	The Oslo Youth Study (OYS)	Norway	*n* = 443	14, 25	1979	Questionnaire	X	N/P	X	X	6
Laska, M. N. *et al.* (2012) 38	Identifying Determinants of Eating and Activity (IDEA) and the Aetiology of Childhood Obesity (ECHO).	US	*n* = 562 to 666	14, 16	IDEA: 2005–2006 ECHO: 2006–2007 (estimated)	Three 24‐h dietary recalls	X	✔	X	X	8
Lee, A. K. *et al.* (2015) 65	National Heart, Lung and Blood Institute Growth Health Study (NGHS)	US	*N* = 840 to 1,304	13, 16	1991	Three day food records	✔	X	X	X	8
Lien, N. *et al.* (2002) 50	The Norwegian Longitudinal Health Behaviour (NLHB) Study	Norway	*n* = 380 to 613	15, 21	1990	Questionnaire	X	D	D	✔	6
Lipsky, L. M. *et al.* (2015) 39	NEXT Generation Health Study (NEXT)	US	*n* = 2,172 to 2,785	16, 17, 18, 19	2006–2007	Questionnaire	X	✔	X	✔	5
Ovrebo, E. M. (2011) 46	—	Norway	*n* = 583 to 606	13, 15	2002	FFQ	X	N/P	N/P	X	7
Patterson, E. *et al.* (2009) 32	European Youth Heart Study (EYHS)	Sweden	*n* = 179, older cohort	15, 21	1996–1997	Single 24‐h dietary recall	✔	✔	✔	X	8
Pearson, N. *et al.* (2011) 44	The Youth Eating Patterns (YEP) study	Australia	*n* = 1,729	13, 15	2004–2005	FFQ	X	✔	X	X	8
Quick, V. *et al.* (2013) 42	Project EAT	US	*n* = 2,134	15, 25	1996–1997	FFQ	X	✔	X	X	7
Stephens, L. D. *et al.* (2014) 51	The Youth Eating Patterns (YEP) study	Australia	*n* = 529, all low SES	12–15, 14–17 (2 year follow‐up)	n/r Baseline assessment in 2004–2005	FFQ	X	D	X	✔	7
Striegel‐Moore, R. H. *et al.* (2006) 40	National Heart, Lung and Blood Institute Growth Health Study (NGHS)	US	*n* = 1944 to 2110, all female	13, 15, 16, 18	1991	Three day food records	X	✔	X	X	7
Thuen, F. *et al.* (2015) 41	The Norwegian Longitudinal Health Behaviour (NLHB) Study	Norway	*n* = 480 to 649	13, 14, 15, 16, 18, 19, 21, 23, 30	1990	Questionnaire	X	✔	X	✔	5
Von Post‐Skagegard, M. *et al.* (2002) 33	—	Sweden	*n* = 208	15, 17, 20	1991–1992	FFQ	X	✔	✔	✔	7
White, J. *et al.* (2014) 66	National Heart, Lung, and Blood Institute Growth Health Study (NGHS)	US	*n* = 774	16, 19	1991	Three day food records	✔	X	X	X	7
Zarrazquin, I. *et al.* (2014) 34	—	Spain	*n* = 285 to 366	19, 20, 21	n/r	FFQ	X	X	N/P	X	6

* Cohort characteristics are only reported here where there is substantial deviation from the general population of that country, e.g. all participants are of a single sex or ethnicity.

For each outcome: X = not reported, D = duplicate: another paper from the same study was selected as the primary paper to represent that study, ✔ = included, G = included in graphs but not meta‐analysis, N/P = data extraction or data conversion not possible.

FFQ, food‐frequency questionnaire; n/r, not reported; US, United States of America.

### Intake of added sugar or sucrose

**Figure 2:**
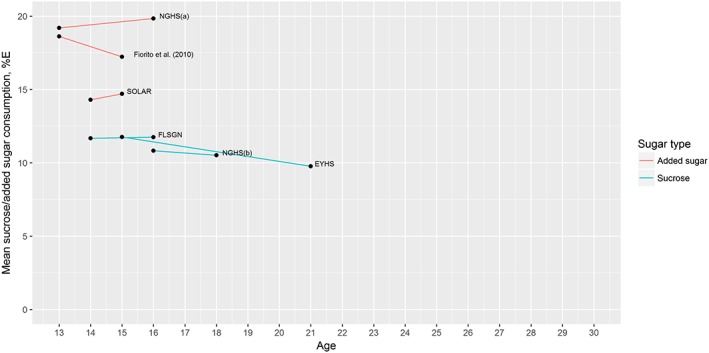
Change in percentage energy intake from added sugar or sucrose by age across five cohorts. [Colour figure can be viewed at wileyonlinelibrary.com]

Six papers reported on intake of added sugar or sucrose intake from five different studies, as shown in Table 2. Across all studies included, except European Youth Heart Study (EYHS) at age 21, mean added sugar/sucrose intake was above the WHO recommendation of less than 10% of total energy intake 10. As evident from Fig. 2, which shows the mean sucrose or added sugar intake as a percentage of total energy intake from each cohort, the data available reported on intake in adolescence only, with no data available beyond the age of 21 years.

Meta‐analysis of the change in intake per year from each study suggests an overall decrease in added sugar and sucrose intake over this period; however, this effect was not significant at the *p* < 0.05 level; see Table 3 for summary effects and the supporting information (Figs S1 to S3) for forest plots of meta‐analyses. Due to the low number of studies, meta‐regression and funnel plot analysis of bias were not conducted. *Post‐hoc* analyses of added sugars and sucrose as separate outcomes found a stronger decrease in sucrose consumption (−0.26% [95%CI −0.43 to −0.10]) compared with added sugar consumption (−0.12% [95%CI −0.86 to 0.62]). An overall decrease of −0.26% energy from sucrose per year suggests that over a period of five years adolescents would see a reduction of 1.3% of their total energy intake derived from sucrose.

**Table 3:** Results from meta‐analysis of sucrose or added sugar, SSBs and confectionery consumption

Item	Subgroup	Number of studies	Age range covered (years)	Pooled change in consumption per year	95% CI of pooled change in consumption per year	I^2^ (%)
Added sugar or sucrose	Both added sugar and sucrose	6	13 to 21	−0.15%energy	−0.41, 0.12	57.2
	Added sugar	3	13 to 16	−0.12%energy	−0.86, 0.62	64.0
	Sucrose	3	14 to 21	−0.26%energy	−0.43, −0.10	0.0
SSBs	All	12	13 to 30	−0.15 servings/week	−0.32 to 0.02	99.7
	Restricted definition of SSBs	8	13 to 30	−0.23 servings/week	−0.42 to −0.04	99.8
	Adolescent	10	13 to 18	−0.08 servings/week	−0.42 to 0.27	99.3
	Early adult	2	18 to 30	−0.46 servings/week	−1.32 to 0.40	61.1
Confectionery	All	5	13 to 23	−0.20 servings/week	−0.41 to −0.001	92.8

### Sugar‐sweetened beverages

**Figure 3:**
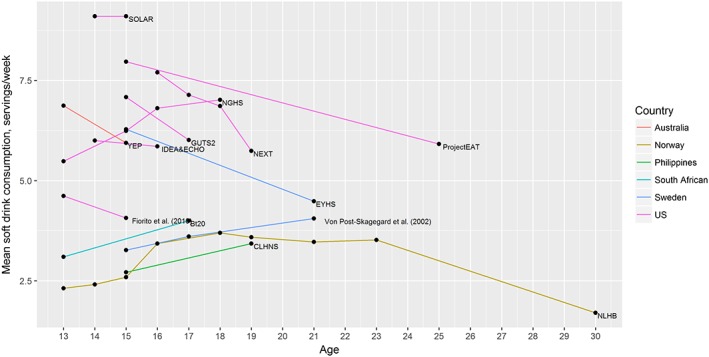
Mean intake of SSBs by age across 13 cohorts. [Colour figure can be viewed at wileyonlinelibrary.com]

Twenty papers reported on SSB intake, reporting on 17 independent studies. Of these, we were able to extract comparable data from 13 studies; the remaining four studies did not report data in a way that could be converted to the standard metric of servings per week, for example reporting proportion of participants above or below a given level of intake rather than average levels of intake. The mean data from the 13 included studies is illustrated in Fig. 3.

Of these 13 studies, one could not be included in the meta‐analysis 36, as no data was provided on the variance of SSB intake among the population. Table 3 shows the results from the meta‐analysis of the remaining 12 studies. Meta‐analysis of these 12 studies showed an overall decrease in SSB consumption over the adolescence and early adulthood period. However, this was not significant at the *p* < 0.05 level (Table 3). Funnel plot asymmetry and Eggers test for bias showed no evidence of asymmetry among included studies of SSB consumption (*p* = 0.52).

Data on SSBs included both studies that used terms such as ‘soft drinks’ and ‘fruit drinks’ and did not specify whether drinks were sugar‐sweetened or artificially sweetened, as well as studies that used a more precise definition of SSBs. As a sensitivity analysis, we included only those studies that precisely defined SSBs (as containing added sugar) or where data on SSBs (precisely defined) were available separately from other categories of soft drinks that were less clearly defined 37, 38, 39, 40, 41, 42, 43, 44

As shown in Table 3, we found a stronger effect when using the stricter definition of SSBs, with a significant decrease in consumption of −0.23 (95%CI −0.42 to −0.04) servings per year of age. This effect size suggests that over 5 years those in this age range would consume 1.15 fewer servings of SSBs each week.

Using all SSBs as the outcome, a sub‐group analysis by gender looked at studies where changes were reported separately for boys and girls. We saw a slightly larger decrease in weekly servings of SSBs per year for girls (−0.20 servings/week, 95%CI −0.41 to 0.02) than for boys (−0.14 servings/week, 95%CI −0.41 to 0.12). However, changes for both genders remained non‐significant at the *p* = 0.05 level.

Meta‐regression to further test for causes of study heterogeneity showed no significant moderation of the effect estimates by study quality or method of diet data collection. Inclusion of the year of study mid‐point in a meta‐regression (to test for a moderation of the effect by calendar time) showed a small but significant association of calendar year with change in consumption seen with age, suggesting that with each calendar year the change in consumption per year of age becomes more negative by 0.04 servings/week (beta = −0.04 servings/week, 95%CI −0.07 to −0.01).

Meta‐regression further assessed whether the effect was moderated by the age range covered by the cohort, using the age that fell at the mid‐point of each cohort age range. However, no significant effect was seen for inclusion of mid‐point age in the meta‐regression. We tested whether a stronger effect was seen if we looked at data only from adolescence (age 13 to 18) or from early adulthood (age 18 to 30). While changes in intake were non‐significant across both age ranges when the data were split in this way (Table 3), the findings suggest that the decreases in consumption may be greater in early adulthood than adolescence. However, the low number of studies with data in early adulthood prevents us from drawing these conclusions with confidence.

Of studies where data could not be included in the meta‐analysis, there was no consistent direction of change in SSB consumption, with one study reporting a decrease in consumption 45, two an increase in consumption 36, 46 and two studies reporting little change or conflicting changes between groups 3, 47.

### Confectionery

**Figure 4:**
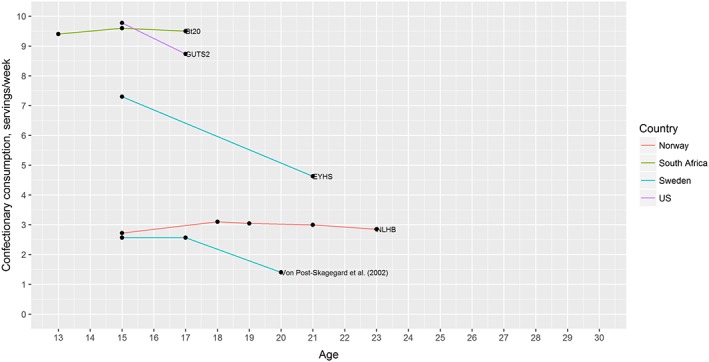
Mean intake of confectionery by age across five cohorts. [Colour figure can be viewed at wileyonlinelibrary.com]

Nine papers reported on confectionery intake, reporting on eight independent studies. Of these, we were able to include five studies in the quantitative analyses. The remaining three studies did not report data in a way that could be converted to the standard metric of servings per week. The mean data from the five included studies are illustrated in Fig. 4.

There is some variability in the definition of what is included in the studies under confectionery, with two studies including a wider range of sugary food items 37, 48, while two studies report on only sweets and chocolate 32, 49. One further study did not document what was included under the term confectionery 33. Meta‐analysis of the five included studies shows a decrease in confectionery consumption over the adolescence and early adulthood period. The pooled effect size of −0.20 (95%CI −0.41 to −0.001) servings/week per year of age suggests that over five years those in this age range would consume one fewer serving of confectionery each week. Due to the low number of studies, meta‐regression and funnel plot analysis of bias were not conducted.

Of the three studies that could not be included in the meta‐analysis, there was no consistency in the data on change in confectionery consumption. Two studies reported decreases in consumption 34, 47, while another reported a significant increase 46 in confectionery consumption.

### Determinants of change in added sugar consumption

Six papers from five studies reported data on determinants of changes in added sugar consumption. Studies reported no effects of gender 33, 39, 50, race or ethnicity 39, mother's education level 33, SES 50 or baseline weight status 39 on changes in intake of SSBs or confectionery. Lien *et al.* (2002) investigated the association of personal, social (family and friends) and environmental (school) factors with change in sugary food and drink consumption, reporting no significant associations of these factors with intake at follow‐up, controlling for baseline intake 50. A further study found no significant effect of family structure on change in SSB consumption 41. However, a number of studies reported associations between change in consumption and a range of behavioural determinants. Screen time at baseline and positive change in screen time was found to positively predict change in consumption of SSBs and sweets over a 2‐year period 37. Stephens *et al.* (2014) reported that less frequent consumption of fast food at baseline predicted less frequent consumption of high‐energy beverages at follow‐up, controlling for baseline consumption 51, as well as an inverse association between availability of high energy foods in the home environment and change in SSB consumption 51. Lien *et al.* (2002) reported no effect of dieting on consumption of confectionery and SSBs at follow‐up, controlling for baseline consumption 50.

## Discussion

### Principal findings

Meta‐analysis of available longitudinal data on intakes of total added sugar or sucrose, SSBs and confectionery together indicate a decrease in added sugar intake across adolescence and into early adulthood. While not all results from the meta‐analysis are significant at the *p* = 0.05 level, the consistent direction of change across nutrients and food groups suggests an overall trend towards decreases in added sugar intake over this age range. The pooled estimates of change in intake per year are small (annual change of −0.12% energy from added sugar (n.s), −0.26% energy from sucrose, −0.20 servings/week confectionery, −0.15 servings/week SSBs (n.s.)), but if maintained through the adolescence and early adulthood years these changes will make a positive contribution towards a more healthy diet. A very small number of papers reported on determinants of changes in added sugar intake, suggesting no association of time‐invariant sociodemographic factors with changes in consumption of sugary foods or drinks, but some association of other health behaviours with such changes.

### Strengths and limitations of this review

To our knowledge this is the first study to systematically review and meta‐analyse data on changes in added sugar intake from adolescence to early adulthood. Our review found sufficient reported data to conduct meta‐analysis across three domains of dietary data: nutrient level data on added sugar or sucrose intake and data on two food groups high in added sugar (SSBs and confectionery). The pooled effects were all in a consistent direction, lending some confidence to our conclusion that added sugar intake decreases across this age range.

Nevertheless, this review was limited by the data available in the reviewed papers and the quality of those papers. While in our review, we intended to look at change in intake across the period from ages 13 to 30, much of the data retrieved was from the adolescent years, with very few reports of studies including data collection in the late twenties. Thus, our findings reported in this review predominantly represent dietary change in adolescence alone. In addition, the majority of studies reviewed came from high income countries and the findings may not be generalizable to low and middle income countries. The limited evidence available on soft drink intake in the later part of the age range of interest suggested that the decrease in soft drink intake may be greater from age 18 to 30 than ages 13 to 18, which might suggest a stronger decrease in overall added sugar intake. However, these adult data came from only two studies, and the pooled effect was non‐significant.

We found limited nutritional data on added sugar intake, with only three studies reporting on total dietary added sugar. Due to the low number of studies reporting on dietary sugar intake, we initially analysed together two different measures of dietary sugar: total added sugar, and total sucrose intake, both as a percentage of overall energy intake. These are partially overlapping but distinct categories of sugar; added sugar includes sucrose but also other sugars, for example high fructose corn syrup 52. Meanwhile sucrose is found in natural sources as well as added to foods in its purified form 53. Results suggested moderate heterogeneity between these six studies. A post‐hoc analysis of studies of added sugar and sucrose separately found a stronger (and significant) decline in sucrose intake compared with added sugar intake; however, the reasons for this effect are unclear. In recent years, sucrose represents a decreasing proportion of all added sugars, with high fructose corn syrup reported to now represent close to one‐half of all caloric sweeteners consumed in the US 52; thus, changes in intake may to some extent reflect secular changes in food composition. In addition, the studies reporting on sucrose include data covering participants at older ages than those reporting on added sugar, so this effect may reflect a stronger decrease in sugar consumption in early adulthood than adolescence, similar to that suggested by our SSB analysis. However, the limited data available in this study do not allow us to explore these hypotheses further.

While more data were available on specific food items (SSBs and confectionery), our meta‐analysis of SSB and confectionery intakes shows a high degree of heterogeneity between studies, which could not be explained by the variables tested through meta‐regression. While this heterogeneity is to some extent accounted for by use of random effects meta‐analysis, such a high degree of heterogeneity suggests that pooled results should be interpreted with caution. Heterogeneity may be due to true differences between study populations, such as social and environmental factors, or may be due to methodological differences whereby studies suffer from different degrees of bias. Indeed, there was considerable variability of data on SSB and confectionery intakes, with differences in data collection, classification and grouping of foods, and units of reporting. Conversions were required in many cases between the reported data and the common units used for meta‐analysis. Some of the heterogeneity between studies may be due to differences in definitions of foods and drinks included in each food group. In several papers, the exact definition of the foods or drinks that were included in the data reported was unclear. However, this lack of precision is more likely to have weakened our findings than produced erroneous findings. In our meta‐analysis of SSB data, we found a larger decrease in intake when including only studies that clearly defined drinks as containing sugar. If some of the studies that were excluded using this stricter definition did indeed include some data on diet drinks, the difference in effect size may reflect a tendency for adolescents to switch from sugar‐sweetened to diet drinks as they get older, as suggested by cross‐sectional data 54.

One consistent limitation of dietary data is the potential for bias introduced through misreporting. However, when looking at change over time, the estimate of change may still be valid if the level of misreporting is constant over time. However, if misreporting changed over the course of the study, either as a result of increasing age of participants, or over time due to changes in public awareness of the health effects of particular food groups, this will have affected our measures of change. While this is difficult to assess, we are reassured that we did not see any of effects of data collection method on the overall effect estimate of change in SSB intake in meta‐regression.

### Relationship of our findings to prior knowledge

Our findings across several related outcomes suggest a small but significant reduction in consumption of added sugar through adolescence and into early adulthood. The overall decrease in added sugar intake over this age range corresponds well with experimental studies of taste preferences. These have shown that young adults have a higher sensitivity towards sucrose than adolescents and young adults have lower preferred optimal sucrose concentrations in foods than adolescents 55, suggesting a universal physiological mechanism, accounting for a decreased preference for sweet tasting foods over this age range.

The reported levels of consumption from the studies included in our review are in line with nationally representative and cross‐national survey data 13, 14, 56, 57, 58. However, the population means of added sugar consumption remained above the WHO recommendation of less than 10% of total energy intake 10 and SSB intake was high, particularly in US studies. Considerable further reduction of intake of added sugars than our pooled estimates suggest will be required to comply with the WHO recommendation.

It is difficult using the longitudinal cohort data reviewed here to disentangle trends due to the increasing age of the cohorts and secular trends. Cross‐sectional data from the US suggest that between 2000 and 2010 there was a secular decrease in intake of added sugar among children and adolescents, including that from SSBs, of around 25% 14, 59. However, prior to this, children's and adolescents' SSB intake is reported to have increased from 204 to 224 kcal day^−1^ from 1988 to 1994 to 1999–2004 60. Meanwhile, intake of non‐milk extrinsic sugars among adolescents in the north east of England was reported to have changed little from 1980 to 2000 61. In our meta‐regression of change in SSB intake, the significant negative effect of calendar year on the effect estimate indicates a greater decrease in SSB intake with each additional calendar year, which may be indicative of an additive effect of a negative secular change in more recent years and a decreasing intake with age.

### Implications for policy, practice and research

This review and meta‐analysis of longitudinal changes in added sugar consumption suggest a consistent trend across sugar categories for a decrease in consumption through adolescence and early adulthood. However, given the small size of the effects seen, this finding does not suggest that the high added sugar consumption in children will reduce to a healthy level by adulthood without further intervention. By contrast, the natural tendency to reduce added sugar intake over this age range may suggest an opportunity for intervention to direct this change in taste towards more healthy foods. School, university or workplace interventions may be helpful to restrict availability of sugary foods and drinks. Alternatively, sugar reduction may form one component of a broader weight control intervention such as those currently being tested by the EARLY consortium 62. More information is needed to determine the most effective way to provide dietary interventions in this age group, as well as studies which look at the relative effect of intervention at different ages.

We identified a lack of data on trajectories of added sugar consumption into early adulthood, with few studies continuing beyond the early twenties. Further longitudinal data in this age range could provide a clearer picture of how changes in added sugar consumption continue into early adulthood. In addition, our analysis here is limited to a study of population means only, which while providing important information about the overall direction of population change, masks variation in trajectories within populations. Further primary data analysis would be useful to further explore variation in trajectories of added sugar intake within populations, as well as factors that contribute to such variation. While few studies included in this review focused on determinants of change in added sugar consumption, the available evidence suggested some association with other health behaviours. Further studies are needed that study in detail interactions between behaviours and explore the potential behavioural determinants of changes in added sugar intake within this age group.

## Funding

The work was undertaken by the Centre for Diet and Activity Research (CEDAR), a UKCRC Public Health Research Centre of Excellence. Funding from the British Heart Foundation, Cancer Research UK, Economic and Social Research Council, Medical Research Council, the National Institute for Health Research (NIHR) and the Wellcome Trust, under the auspices of the UK Clinical Research Collaboration, is gratefully acknowledged. This work was additionally supported by the Medical Research Council [Unit Programme number MC_UU_12015/7]. The views expressed in this paper are those of the authors and not necessarily those of the above named funders.

## Conflict of interest statement

All authors have completed the ICMJE uniform disclosure form at www.icmje.org/coi_disclosure.pdf and declare: all authors had financial support from the above named funders for the submitted work, T.P. additionally declares support from a studentship from the Cambridge Commonwealth Trust; M.W. reports board membership of the MRC, employment by NIHR and grants from NIHR, MRC and the UK Department of Health, E.v.S. declares grants from the UK Department of Health, MRC, NIHR and ESRC; K.C. declares a grant from NIHR; K.C. declares that she is a Director of Ridgepoint Consulting Ltd.

## Supporting information

Table S1: Medline search strategy
Table S2: Data conversion and assumptions for each paper included in graphs and/or meta‐analysis
Table S3: Quality assessment tool for longitudinal observational studies of diet
Figure S1: Forest plot depicting % energy from added sugar or sucrose and study weighting for all studies reporting on change in intake of added sugar or sucrose, together with the combined estimate.
Figure S2: Forest plot depicting servings/day and study weighting for all studies reporting on change in intake of SSBs, together with the combined estimate.
Figure S3: Forest plot depicting servings/day and study weighting for all studies reporting on change in intake of confectionery, together with the combined estimate.

## References

[1] Malik VS , Pan A , Willett WC , Hu FB . Sugar‐sweetened beverages and weight gain in children and adults: a systematic review and meta‐analysis. Am J Clin Nutr 2013; 98: 1084–1102. 23966427 10.3945/ajcn.113.058362 PMC3778861

[2] Zheng M , Rangan A , Olsen NJ *et al.* Substituting sugar‐sweetened beverages with water or milk is inversely associated with body fatness development from childhood to adolescence. Nutrition 2015; 31: 38–44. 25441586 10.1016/j.nut.2014.04.017

[3] Ambrosini GL , Oddy WH , Huang RC , Mori TA , Beilin LJ , Jebb SA . Prospective associations between sugar‐sweetened beverage intakes and cardiometabolic risk factors in adolescents. Am J Clin Nutr 2013; 98: 327–334. 23719557 10.3945/ajcn.112.051383 PMC3712546

[4] Craigie AM , Lake AA , Kelly SA , Adamson AJ , Mathers JC . Tracking of obesity‐related behaviours from childhood to adulthood: a systematic review. Maturitas 2011; 70: 266–284. 21920682 10.1016/j.maturitas.2011.08.005

[5] Imamura F , O'Connor L , Ye Z *et al.* Consumption of sugar sweetened beverages, artificially sweetened beverages, and fruit juice and incidence of type 2 diabetes: systematic review, meta‐analysis, and estimation of population attributable fraction. BMJ 2015; 351: h3576. 26199070 10.1136/bmj.h3576 PMC4510779

[6] Basu S , McKee M , Galea G , Stuckler D . Relationship of soft drink consumption to global overweight, obesity, and diabetes: a cross‐national analysis of 75 countries. Am J Public Health 2013; 103: 2071–2077. 23488503 10.2105/AJPH.2012.300974 PMC3828681

[7] Malik VS , Popkin BM , Bray GA , Despres JP , Willett WC , Hu FB . Sugar‐sweetened beverages and risk of metabolic syndrome and type 2 diabetes: a meta‐analysis. Diabetes Care 2010; 33: 2477–2481. 20693348 10.2337/dc10-1079 PMC2963518

[8] Vartanian LR , Schwartz MB , Brownell KD . Effects of soft drink consumption on nutrition and health: a systematic review and meta‐analysis. Am J Public Health 2007; 97: 667–675. 17329656 10.2105/AJPH.2005.083782 PMC1829363

[9] Yang Q , Zhang Z , Gregg EW , Flanders WD , Merritt R , Hu FB . Added sugar intake and cardiovascular diseases mortality among US adults. JAMA Intern Med 2014; 174: 516–524. 24493081 10.1001/jamainternmed.2013.13563 PMC10910551

[10] Anon . Guideline: “Sugars Intake for Adults and Children.”. World Health Organization: Geneva, 2015. 25905159

[11] Sluik D , van Lee L , Engelen AI , Feskens EJM . Total, free, and added sugar consumption and adherence to guidelines: the Dutch National Food Consumption Survey 2007–2010. Nutrients 2016; 8: 70. 26828518 10.3390/nu8020070 PMC4772034

[12] Louie JCY , Moshtaghian H , Rangan AM , Flood VM , Gill TP . Intake and sources of added sugars among Australian children and adolescents. Eur J Nutr 2015; 55: 2347–2355. 26377592 10.1007/s00394-015-1041-8

[13] Gibson S , Francis L , Newens K , Livingstone B . Associations between free sugars and nutrient intakes among children and adolescents in the UK. Br J Nutr 2016: 1–10. 10.1017/S0007114516003184 27641637

[14] Welsh JA , Sharma AJ , Grellinger L , Vos MB . Consumption of added sugars is decreasing in the United States. Am J Clin Nutr 2011; 94: 726–734. 21753067 10.3945/ajcn.111.018366 PMC3155936

[15] Brisbois TD , Marsden SL , Harvey Anderson G , Sievenpiper JL . Estimated intakes and sources of total and added sugars in the Canadian diet. Nutrients 2014; 6: 1899–1912. 24815507 10.3390/nu6051899 PMC4042566

[16] Nelson MC , Story M , Larson NI , Neumark‐Sztainer D , Lytle LA . Emerging adulthood and college‐aged youth: an overlooked age for weight‐related behavior change. Obesity 2008; 16: 2205–2211. 18719665 10.1038/oby.2008.365

[17] Viner RM , Ross D , Hardy R *et al.* Life course epidemiology: recognising the importance of adolescence. J Epidemiol Community Health 2015; 69: 719–720. 25646208 10.1136/jech-2014-205300 PMC4515995

[18] Rindfuss RR . The young adult years: diversity, structural change, and fertility. Demography 1991; 28: 493–512. 1769399

[19] Verplanken B , Walker I , Davis A , Jurasek M . Context change and travel mode choice: combining the habit discontinuity and self‐activation hypotheses. J Environ Psychol 2008; 28: 121–127.

[20] Verplanken B , Roy D . Empowering interventions to promote sustainable lifestyles: testing the habit discontinuity hypothesis in a field experiment. J Environ Psychol 2016; 45: 127–134.

[21] Emmett PM , Jones LR . Diet, growth, and obesity development throughout childhood in the Avon Longitudinal Study of Parents and Children. Nutr Rev 2015; 73: 175–206. 10.1093/nutrit/nuv054 PMC4586450 26395342

[22] Nelson MC , Neumark‐Sztainer D , Hannan PJ , Story M . Five‐year longitudinal and secular shifts in adolescent beverage intake: findings from project EAT (Eating Among Teens)‐II. J Am Diet Assoc 2009; 109: 308–312. 19167959 10.1016/j.jada.2008.10.043

[23] Winpenny EM , Penney TL , Corder K , White M , van Sluijs EMF . Change in diet in the period from adolescence to early adulthood: a systematic scoping review of longitudinal studies. Int J Behav Nutr Phys Act. 10.1186/s12966-017-0518-7 PMC5418762 28472992

[24] Arnett JJ . Emerging adulthood: a theory of development from the late teens through the twenties. Am Psychol 2000; 55: 469–480. 10842426

[25] Singh a S , Mulder C , Twisk JWR , Van Mechelen W , Chinapaw MJM . Tracking of childhood overweight into adulthood: a systematic review of the literature. Obes Rev 2008; 9: 474–488. 18331423 10.1111/j.1467-789X.2008.00475.x

[26] Jones RA , Hinkley T , Okely AD , Salmon J . Tracking physical activity and sedentary behavior in childhood: a systematic review. Am J Prev Med 2013; 44: 651–658. 23683983 10.1016/j.amepre.2013.03.001

[27] Tanaka C , Reilly JJ , Huang WY . Longitudinal changes in objectively measured sedentary behaviour and their relationship with adiposity in children and adolescents: systematic review and evidence appraisal. Obes Rev 2014; 15: 791–803. 24899125 10.1111/obr.12195

[28] Tooth L , Ware R , Bain C , Purdie DM , Dobson A . Quality of reporting of observational longitudinal research. Am J Epidemiol 2005; 161: 280–288. 15671260 10.1093/aje/kwi042

[29] Higgins J, Green S eds. Cochrane Handbook for Systematic Reviews of Interventions. Version 5. The Cochrane Collaboration; 2011 http://handbook-5-1.cochrane.org/

[30] Egger M , Davey Smith G , Schneider M , Minder C . Bias in meta‐analysis detected by a simple, graphical test. Br Med J 1997; 315: 629–634. 9310563 10.1136/bmj.315.7109.629 PMC2127453

[31] Moher D , Liberati A , Tetzlaff J , Altman DG , Grp P . Preferred Reporting Items for Systematic Reviews and Meta‐Analyses: the PRISMA Statement (Reprinted from Annals of Internal Medicine). Phys Ther 2009; 89: 873–880. 19723669

[32] Patterson E , Wärnberg J , Kearney J , Sjöström M . The tracking of dietary intakes of children and adolescents in Sweden over six years: the European Youth Heart Study. Int J Behav Nutr Phys Act 2009; 6. 10.1186/1479-5868-6-91 PMC2797763 20003331

[33] von Post‐Skagegård M , Samuelson G , Karlström B , Mohsen R , Berglund L , Bratteby L‐E . Changes in food habits in healthy Swedish adolescents during the transition from adolescence to adulthood. Eur J Clin Nutr 2002; 56: 532–538. 12032653 10.1038/sj.ejcn.1601345

[34] Zarrazquin I , Torres‐Unda J , Ruiz F *et al.* Longitudinal study: lifestyle and cardiovascular health in health science students. Nutr Hosp 2014; 30: 1144–1151. 25365020 10.3305/nh.2014.30.5.7833

[35] Magarey A , Watson J , Golley RK *et al.* Assessing dietary intake in children and adolescents: considerations and recommendations for obesity research. Int J Pediatr Obes 2011; 6: 2–11. 10.3109/17477161003728469 20874449

[36] Adair LS , Popkin BM . Are child eating patterns being transformed globally? Obes Res 2005; 13: 1281–1299. 16077000 10.1038/oby.2005.153

[37] Falbe J , Willett WC , Rosner B , Gortmaker SL , Sonneville KR , Field AE . Longitudinal relations of television, electronic games, and digital versatile discs with changes in diet in adolescents. Am J Clin Nutr 2014; 100: 1173–1181. 25240080 10.3945/ajcn.114.088500 PMC4163796

[38] Laska MN , Murray DM , Lytle LA , Harnack LJ . Longitudinal associations between key dietary behaviors and weight gain over time: transitions through the adolescent years. Obesity 2012; 20: 118–125. 21701567 10.1038/oby.2011.179 PMC3402912

[39] Lipsky LM , Haynie DL , Liu D *et al.* Trajectories of eating behaviors in a nationally representative cohort of U.S. adolescents during the transition to young adulthood. Int J Behav Nutr Phys Act 2015; 12: 138. 26537771 10.1186/s12966-015-0298-x PMC4632654

[40] Striegel‐Moore RH , Thompson D , Affenito SG *et al.* Correlates of beverage intake in adolescent girls: the National Heart, Lung, and Blood Institute Growth and Health Study. J Pediatr 2006; 148: 183–187. 16492426 10.1016/j.jpeds.2005.11.025

[41] Thuen F , Breivik K , Wold B , Ulveseter G . Growing up with one or both parents: the effects on physical health and health‐related behavior through adolescence and into early adulthood. J Divorce Remarriage 2015; 56: 451–474.

[42] Quick V , Wall M , Larson N , Haines J , Neumark‐Sztainer D . Personal, behavioral and socio‐environmental predictors of overweight incidence in young adults: 10‐yr longitudinal findings. Int J Behav Nutr Phys Act 2013; 10: 37. 23531253 10.1186/1479-5868-10-37 PMC3623851

[43] Davis JN , Alexander KE , Ventura EE , Toledo‐Corral CM , Goran MI . Inverse relation between dietary fiber intake and visceral adiposity in overweight Latino youth. Am J Clin Nutr 2009; 90: 1160–1166. 19793854 10.3945/ajcn.2009.28133 PMC2762155

[44] Pearson N , Ball K , Crawford D . Mediators of longitudinal associations between television viewing and eating behaviours in adolescents. Int J Behav Nutr Phys Act 2011; 8: 23. 21450065 10.1186/1479-5868-8-23 PMC3078829

[45] Kvaavik E , Andersen LF , Klepp K‐I . The stability of soft drinks intake from adolescence to adult age and the association between long‐term consumption of soft drinks and lifestyle factors and body weight. Public Health Nutr 2005; 8: 149–157. 15877908 10.1079/phn2004669

[46] Øvrebø EM . Food habits of school pupils in Tromso, Norway, in the transition from 13 to 15 years of age. Int J Consum Stud 2011; 35: 520–528.

[47] Bruno‐Ambrosius K , Swanholm G , Twetman S . Eating habits, smoking and toothbrushing in relation to dental caries: a 3‐year study in Swedish female teenagers. Int J Paediatr Dent 2005; 15: 190–196. 15854115 10.1111/j.1365-263X.2005.00621.x

[48] Feeley A , Musenge E , Pettifor JM , Norris SA . Changes in dietary habits and eating practices in adolescents living in urban South Africa: the birth to twenty cohort. Nutrition 2012; 28: e1–e6. 22465902 10.1016/j.nut.2011.11.025

[49] Åstrøm AN . Stability of oral health‐related behaviour in a Norwegian cohort between the ages of 15 and 23 years. Community Dent Oral Epidemiol 2004; 32: 354–362. 15341620 10.1111/j.1600-0528.2004.00174.x

[50] Lien N , Jacobs DR , Klepp K‐I . Exploring predictors of eating behaviour among adolescents by gender and socio‐economic status. Public Health Nutr 2002; 5: 671–681. 12372162 10.1079/PHN2002334

[51] Stephens LD , McNaughton SA , Crawford D , Ball K . Predictors of high‐energy foods and beverages: a longitudinal study among socio‐economically disadvantaged adolescents. Public Health Nutr 2014; 17: 324–337. 23122445 10.1017/S136898001200482X PMC10282232

[52] Bray GA , Nielsen SJ , Popkin BM . Consumption of high‐fructose corn syrup in beverages may play a role in the epidemic of obesity. Am J Clin Nutr 2004; 79: 537–543. 15051594 10.1093/ajcn/79.4.537

[53] Erickson J , Slavin J . Total, added, and free sugars: are restrictive guidelines science – based or achievable? Nutrients 2015; 7: 2866–2878. 25884659 10.3390/nu7042866 PMC4425178

[54] Fakhouri THI , Kit BK , Ogden CL . Consumption of diet drinks in the United States, 2009–2010. NCHS data Br No 2012; 109: 1–8. 23102235

[55] De Graaf C , Zandstra EH . Sweetness intensity and pleasantness in children, adolescents, and adults. Physiol Behav 1999; 67: 513–520. 10549887 10.1016/s0031-9384(99)00090-6

[56] Public Health England. *National Diet and Nutrition Survey Results from Years 5 and 6 (combined) of the Rolling Programme (2012/2013–2013/2014)* London; 2016.

[57] Public Health England and Food Standards Agency. National Diet and Nutrition Survey: results from Years 1 to 4 (combined) of the rolling programme for 2008 and 2009 to 2011 and 2012. 2014.

[58] Duffey KJ , Huybrechts I , Mouratidou T *et al.* Beverage consumption among European adolescents in the HELENA study. Eur J Clin Nutr 2012; 66: 244–252. 21952695 10.1038/ejcn.2011.166 PMC3392586

[59] Mesirow MSC , Welsh JA . Changing beverage consumption patterns have resulted in fewer liquid calories in the diets of US children: National Health and Nutrition Examination Survey 2001–2010. J Acad Nutr Diet 2015; 115: 559–566. 25441966 10.1016/j.jand.2014.09.004

[60] Wang YC , Bleich SN , Gortmaker SL . Increasing caloric contribution from sugar‐sweetened beverages and 100% fruit juices among US children and adolescents, 1988–2004. Pediatrics 2008; 121: e1604–e1614. 18519465 10.1542/peds.2007-2834

[61] Rugg‐Gunn AJ , Fletcher ES , Matthews JNS *et al.* Changes in consumption of sugars by English adolescents over 20 years. Public Health Nutr 2007; 10: 354–363. 17362531 10.1017/S1368980007249729

[62] Lytle LA , Svetkey LP , Patrick K *et al.* The EARLY trials: a consortium of studies targeting weight control in young adults. Transl Behav Med 2014; 4: 304–313. 25264469 10.1007/s13142-014-0252-5 PMC4167899

[63] Deheeger M , Bellisle F , Rolland‐Cachera MF . The French longitudinal study of growth and nutrition: data in adolescent males and females. J Hum Nutr Diet 2002; 15: 429–438. 12460151 10.1046/j.1365-277x.2002.00396.x

[64] Fiorito LM , Marini M , Mitchell DC , Smiciklas‐Wright H , Birch LL . Girls' early sweetened carbonated beverage intake predicts different patterns of beverage and nutrient intake across childhood and adolescence. J Am Diet Assoc 2010; 110: 543–550. 20338280 10.1016/j.jada.2009.12.027

[65] Lee AK , Chowdhury R , Welsh JA . Sugars and adiposity: the long‐term effects of consuming added and naturally occurring sugars in foods and in beverages. Obes Sci Pract 2015; 1: 41–49. 27774248 10.1002/osp4.7 PMC5057365

[66] White J , Jago R , Thompson JL . Dietary risk factors for the development of insulin resistance in adolescent girls: a 3‐year prospective study. Public Health Nutr 2014; 17: 361–368. 23158020 10.1017/S1368980012004983 PMC10282440

