# Supplementary material for: Changes in consumption of added sugars from age 13 to 30 years: a systematic review and meta‐analysis of longitudinal studies
Source: Obes Rev. 2017 Sep 4;18(11):1336–49. doi: 10.1111/obr.12588 (PMC5656815; doi:10.1111/obr.12588)

Changes in consumption of added sugars from age 13 to 30 years: a systematic review and meta-analysis of longitudinal studies

Eleanor M Winpenny, Tarra L Penney, Kirsten Corder, Martin White, Esther MF van Sluijs

Table S1: Medline search strategy

1	Diet Outcomes Physical activity outcomes	food*[Title/Abstract] OR nutrient*[Title/Abstract] OR macronutrient*[Title/Abstract] OR "energy intake"[Title/Abstract] OR diet[Title/Abstract] OR diets[Title/Abstract] OR "dietary"[Title/Abstract] OR nutrition[Title/Abstract] OR nutritional[Title/Abstract] OR fruit[Title/Abstract] OR vegetable[Title/Abstract] OR fruits[Title/Abstract] OR vegetables[Title/Abstract] OR snack*[Title/Abstract] OR "soft drink*"[Title/Abstract] OR soda[Title/Abstract] OR SSB[Title/Abstract] OR SSBs[Title/Abstract] OR salt[Title/Abstract] OR sugar*[Title/Abstract] OR "Food"[Mesh] OR "Beverages"[Mesh] OR diet[Mesh] OR "Nutrition Surveys"[Mesh] OR "Diet Records"[Mesh] OR "Dietary Fats"[Mesh] OR "Dietary Proteins"[Mesh] OR "Dietary Carbohydrates "[Mesh] OR "Micronutrients"[Mesh] OR "Exercise"[MeSH] OR "Sports"[Mesh] OR "physical activity"[Title/Abstract] OR "physical activities"[Title/Abstract] OR "physically active"[Title/Abstract] OR "active transport"[Title/Abstract] OR "active travel"[Title/Abstract] OR exercise*[Title/Abstract] OR cycle[Title/Abstract] OR cycling[Title/Abstract] OR walk*[Title/Abstract] OR sport*[Title/Abstract] OR "energy expenditure"[Title/Abstract]
2	Longitudinal	longitudinal[Title/Abstract] OR cohort[Title/Abstract] OR prospective[Title/Abstract] OR "follow-up stud*"[Title/Abstract] OR "follow up stud*"[Title/Abstract] OR tracking[Title/Abstract] OR "Follow-Up Studies"[Mesh] OR "Prospective Studies"[Mesh] OR "Longitudinal Studies"[Mesh]
3	Age range	adolescent*[Title/Abstract] OR adolescence[Title/Abstract] OR teen*[Title/Abstract] OR student*[Title/Abstract] OR "young adult*"[Title/Abstract] OR "young adulthood"[Title/Abstract] OR "early adulthood"[Title/Abstract] OR "emerging adulthood"[Title/Abstract] OR youth*[Title/Abstract] OR "young people"[Title/Abstract] OR freshman[Title/Abstract] OR freshmen[Title/Abstract]
4	Additional filters	English[lang]<br>Restrict to publication year 1980 or after
5		1 AND 2 AND 3 AND 4

Note: This search strategy was designed for the initial scoping review of this study [1], which included a focus on physical activity as well as other dietary behaviours.

<u>References</u>

1. Winpenny EM, Penney TL, Corder K, White M, van Sluijs EMF. Change in diet in the period from adolescence to early adulthood: a systematic scoping review of longitudinal studies. Int J Behav Nutr Phys Act. (under revision)

Table S2: Data conversion and assumptions for each paper included in graphs and/or meta-analysis

Reference	Study	Conversions	Assumptions
Added sugar or sucrose			
Davis, J. N., K. E. Alexander, E. E. Ventura, C. M. Toledo-Corral and M. I. Goran (2009). "Inverse relation between dietary fiber intake and visceral adiposity in overweight Latino youth." Am J Clin Nutr 90: 1160-1166.	SOLAR (Study of Latino Adolescents at Risk for Diabetes) cohort	None	
Deheeger, M., F. Bellisle and M. F. Rolland-Cachera (2002). "The French longitudinal study of growth and nutrition: data in adolescent males and females." J Hum Nutr Diet 15: 429-438.	The French longitudinal study of growth and nutrition	Data merged across 2 groups: gender	
Fiorito, L. M., M. Marini, D. C. Mitchell, H. Smiciklas-Wright and L. L. Birch (2010). "Girls' early sweetened carbonated beverage intake predicts different patterns of beverage and nutrient intake across childhood and adolescence." J Am Diet Assoc 110: 543-550.	Fiorito et al. (2010)	Data merged across 2 groups: soda consumers and non-consumers	
Lee AK, Chowdhury R, Welsh JA. Sugars and adiposity: the long-term effects of consuming added and naturally occurring sugars in foods and in beverages. Obes Sci Pract 2015; 1: 41–9.	National Heart, Lung, and Blood Institute Growth Health Study (NGHS)	Tsp of added sugar and total energy used to calculate %energy from added sugar	
Patterson, E., J. Wärnberg, J. Kearney and M. Sjöström (2009). "The tracking of dietary intakes of children and adolescents in Sweden over six years: The European Youth Heart Study." International Journal of Behavioral Nutrition and Physical Activity 6.	European Youth Heart Study	Median and IQR converted to mean and SD	
White, J., R. Jago and J. L. Thompson (2014). "Dietary risk factors for the development of insulin resistance in adolescent girls: a 3-year prospective study." Public Health Nutr 17: 361-368.	National Heart, Lung, and Blood Institute Growth Health Study (NGHS)	g of sucrose and total energy used to calculate %energy from sucrose	
SSBs			
Adair, L. S. and B. M. Popkin (2005). "Are child eating patterns being transformed globally?" Obesity Research 13: 1281-1299.	Cebu Longitudinal Health and Nutrition Study	%energy from soft drinks and total energy used to calculate servings.	Energy content 0.427 kcal/g (NDNS data)<br>NDNS serving size
Davis, J. N., K. E. Alexander, E. E. Ventura, C. M. Toledo-Corral and M. I. Goran (2009). "Inverse relation between dietary fiber intake and visceral adiposity in overweight Latino youth." Am J Clin Nutr 90: 1160-1166.	SOLAR (Study of Latino Adolescents at Risk for Diabetes) cohort	Multiplication x7 for servings/week	
Falbe, J., W. C. Willett, B. Rosner, S. L. Gortmaker, K. R. Sonneville and A. E. Field (2014). "Longitudinal relations of television, electronic games, and digital versatile discs with changes in diet in adolescents." Am J Clin Nutr 100: 1173-1181.	Growing Up Today Study II	Baseline and change data used to calculate follow-up	
Feeley, A., E. Musenge, J. M. Pettifor and S. A. Norris (2012). "Changes in dietary habits and eating practices in adolescents living in urban South Africa: the birth to twenty cohort." Nutrition 28: e1-6.	Birth to Twenty (Bt20) study	None	
Fiorito, L. M., M. Marini, D. C. Mitchell, H. Smiciklas-Wright and L. L. Birch (2010). "Girls' early sweetened carbonated beverage intake predicts different patterns of beverage and nutrient intake across childhood and adolescence." J Am Diet Assoc 110: 543-550.	Fiorito et al. (2010)	Fl oz converted to servings<br>Data merged across 2 drink types	NHANES serving size
Laska, M. N., D. M. Murray, L. A. Lytle and L. J. Harnack (2012). "Longitudinal associations between	Identifying Determinants of	Multiplication x7 for servings/week	

key dietary behaviors and weight gain over time: transitions through the adolescent years." Obesity (Silver Spring) 20: 118-125.	Eating and Activity (IDEA) and the Etiology of Childhood Obesity (ECHO).	Data merged across 2 groups: gender	
Lipsky, L. M., D. L. Haynie, D. P. Liu, A. Chaurasia, B. Gee, K. G. Li, R. J. Iannotti and B. Simons-Morton (2015). "Trajectories of eating behaviors in a nationally representative cohort of US adolescents during the transition to young adulthood." International Journal of Behavioral Nutrition and Physical Activity 12: 11.	NEXT Generation Health Study	None	
Patterson, E., J. Wärnberg, J. Kearney and M. Sjöström (2009). "The tracking of dietary intakes of children and adolescents in Sweden over six years: The European Youth Heart Study." International Journal of Behavioral Nutrition and Physical Activity 6.	European Youth Heart Study	Median, IQR converted to mean, SD<br>Data merged across 2 groups: consumers and non-consumers<br>Grams to servings	NDNS serving size
Pearson, N., K. Ball and D. Crawford (2011). "Mediators of longitudinal associations between television viewing and eating behaviours in adolescents." Int J Behav Nutr Phys Act 8: 23.	The Youth Eating Patterns (YEP) study	Data merged across 2 groups: gender	
Quick, V., M. Wall, N. Larson, J. Haines and D. Neumark-Sztainer (2013). "Personal, behavioral and socio-environmental predictors of overweight incidence in young adults: 10-yr longitudinal findings." Int J Behav Nutr Phys Act 10: 37.	Project EAT 1	Data merged across 2 groups: gender<br>Baseline and change data used to calculate follow-up.	
Striegel-Moore, R. H., D. Thompson, S. G. Affenito, D. L. Franko, E. Obarzanek, B. A. Barton, G. B. Schreiber, S. R. Daniels, M. Schmidt and P. B. Crawford (2006). "Correlates of beverage intake in adolescent girls: the National Heart, Lung, and Blood Institute Growth and Health Study." J Pediatr 148: 183-187.	National Heart, Lung, and Blood Institute Growth Health Study (NGHS)	Grams/day converted to servings/week<br>Data merged across 2 groups: ethnicity<br>Data added across 2 drink types	NHANES serving size
Thuen, F., K. Breivik, B. Wold and G. Ulveseter (2015). "Growing Up with One or Both Parents: The Effects on Physical Health and Health-Related Behavior Through Adolescence and into Early Adulthood." Journal of Divorce and Remarriage 56: 451-474.	The Norwegian Longitudinal Health Behaviour (NLHB) Study	Number in each consumpton response category summed to give overall mean and SD	
Von Post-Skagegard, M., G. Samuelson, B. Karlstrom, R. Mohsen, L. Berglund, L. E. Bratteby, M. von Post-Skagegård, G. Samuelson, B. Karlström, R. Mohsen, L. Berglund and L. E. Bratteby (2002). "Changes in food habits in healthy Swedish adolescents during the transition from adolescence to adulthood." Eur J Clin Nutr 56: 532-538.	Von Post-Skagegard et al. (2002)	Median, IQR converted to mean, SD<br>Data merged across 2 groups: gender	
Confectionery			
Astrom, A. N. and A. N. Åstrøm (2004). "Stability of oral health-related behaviour in a Norwegian cohort between the ages of 15 and 23 years." Community Dent Oral Epidemiol 32: 354-362.	The Norwegian Longitudinal Health Behaviour (NLHB) Study	SD calculated from 95% Cis.<br>Data merged across 2 groups: gender	
Falbe, J., W. C. Willett, B. Rosner, S. L. Gortmaker, K. R. Sonneville and A. E. Field (2014). "Longitudinal relations of television, electronic games, and digital versatile discs with changes in diet in adolescents." Am J Clin Nutr 100: 1173-1181.	Growing Up Today Study II	Data merged across 2 groups: gender<br>Baseline and change data used	

		to calculate follow-up.	
Feeley, A., E. Musenge, J. M. Pettifor and S. A. Norris (2012). "Changes in dietary habits and eating practices in adolescents living in urban South Africa: the birth to twenty cohort." Nutrition 28: e1-6.	Birth to Twenty (Bt20) study	None	
Patterson, E., J. Wärnberg, J. Kearney and M. Sjöström (2009). "The tracking of dietary intakes of children and adolescents in Sweden over six years: The European Youth Heart Study." International Journal of Behavioral Nutrition and Physical Activity 6.	European Youth Heart Study	Median, IQR converted to mean, SD<br>Data merged across 2 groups: consumers and non-consumers<br>Grams to servings	NDNS serving size
Von Post-Skagegard, M., G. Samuelson, B. Karlstrom, R. Mohsen, L. Berglund, L. E. Bratteby, M. von Post-Skagegård, G. Samuelson, B. Karlström, R. Mohsen, L. Berglund and L. E. Bratteby (2002). "Changes in food habits in healthy Swedish adolescents during the transition from adolescence to adulthood." Eur J Clin Nutr 56: 532-538.	Von Post-Skagegard et al. (2002)	Median, IQR converted to mean, SD<br>Multiplication x7 for servings/week	

Further notes on data conversions:

In order to convert from grams or ml to servings we used average serving size data from the US National Health and Nutrition Examination Survey (NHANES) (1999-2010) or the UK National Diet and Nutrition Survey (NDNS; 2008-2012) national survey data [1,2]. For soft drinks, NHANES average serving size (503.25g) was used for 2 US studies [3,4], NDNS average serving size (273.97g) was used for two non-US studies [5,6]. For confectionery, serving size of 33.75g (average serving size from NDNS) was assumed for only one study reported in grams [6]. Where median and interquartile range (IQR) were reported instead of mean and standard deviation (SD), these were converted to mean and SD using formulae 14 and 17 from Wan et al. (2014)[7]. Where data were reported only according to population subgroups, rather than the whole population, these data were combined as recommended by the Cochrane Collaboration [8]. Where sugar-sweetened carbonated drinks and fruit drinks were presented separately, we added these together to produce an overall intake of soft drinks. In order to examine effects against calendar time we used the year when the cohort was mean age 13 and extrapolated to other years. If a range of calendar time was reported, we took that middle of the range given.

<u>References</u>

1. Public Health England and Food Standards Agency. National Diet and Nutrition Survey: results from Years 1 to 4 (combined) of the rolling programme for 2008 and 2009 to 2011 and 2012 [Internet]. 2014. Available: https://www.gov.uk/government/statistics/national-diet-and-nutrition-survey-results-from-years-1-to-4-combined-of-the-rolling-programme-for-2008-and-2009-to-2011-and-2012
2. National Center for Health Statistics. National Health and Nutrition Examination Survey, NHANES (1999-2010) [Internet]. 2016. Available: www.cdc.gov/nchs/nhanes.htm
3. Striegel-Moore RH, Thompson D, Affenito SG, Franko DL, Obarzanek E, Barton BA, et al. Correlates of beverage intake in adolescent girls: the National Heart, Lung, and Blood Institute Growth and Health Study. J Pediatr. 2006;148: 183–7. doi:10.1016/j.jpeds.2005.11.025
4. Fiorito LM, Marini M, Mitchell DC, Smiciklas-Wright H, Birch LL. Girls' Early Sweetened Carbonated Beverage Intake Predicts Different Patterns of Beverage and Nutrient Intake across Childhood and Adolescence. J Am Diet Assoc. Elsevier Inc.; 2010;110: 543–550. doi:10.1016/j.jada.2009.12.027
5. Adair LS, Popkin BM. Are child eating patterns being transformed globally? Obes Res. 2005;13: 1281–99. doi:10.1038/oby.2005.153
6. Patterson E, Wärnberg J, Kearney J, Sjöström M. The tracking of dietary intakes of children and adolescents in Sweden over six years: the European Youth Heart Study. Int J Behav Nutr Phys Act. 2009;6. doi:10.1186/1479-5868-6-91
7. Wan X, Wang W, Liu J, Tong T. Estimating the sample mean and standard deviation from the sample size, median, range and/or interquartile range. BMC Med Res Methodol. 2014;14: 135. doi:10.1186/1471-2288-14-135
8. Higgins J, Green S, editors. Cochrane Handbook for Systematic Reviews of Interventions. Version 5. The Cochrane Collaboration;

Table S3: Quality assessment tool for longitudinal observational studies of diet

Item	Description
A. Study population and participation (baseline): the study sample represents the population of interest on key characteristics.	
1. Adequate description of sampling frame, recruitment methods, period of recruitment and place of recruitment	Item was scored positively if three of the following points were mentioned: (i) description of how participants were sampled (e.g. sourced from the electoral roll, all schools in state or country, or from a larger study); (ii) description of specific methods used for recruitment (e.g. newsletters, phone call, advertising); (iii) period of recruitment provided (e.g. March 2010); or (iv) place of recruitment detailed (e.g. Wollongong, Australia, Glasgow)
2. Adequate description of baseline study sample for key characteristics	Item was scored positively if all three of the following points were mentioned: (i) number of participants; (ii) age (mean age or % at each age); and (iii) gender
B. Study attrition: loss to follow-up not associated with key characteristics (i.e. the study data adequately represent the sample)	
3. Provision of the exact number of participants at each follow-up measurement(s)	Item was scored positively if the number or percentage of participants at each time point was detailed
4. Provision of exact information on follow-up duration	Item was scored positively if detail about the follow-up duration (e.g. 1 year, 6 years) was provided
5. Presentation of data showing non-selective non-response during follow-up measurement(s)	Item was scored positively if those who dropped out of the study were similar on key characteristics to those who were retained at follow-up.
C. Data collection	
6. Adequate description of methods of diet data collection (i.e. tools and processes)	Comprehensive measurement of diet: item was scored positively if both of the following points were mentioned: (i) type of instrument; (ii) number of days measured.
	Questionnaire measures: Item was scored *positively* if the questions used and response options were described in full.
7. Comprehensive diet measurement method	Item was scored positively if a comprehensive diet measurement instrument (e.g. diet record, 24-hr recall, FFQ) was used. 0 points scored for any other method e.g. questions in a questionnaire

8. Adjustment for mis-reporting	Item was scored positively if a description of adjustment for mis-reporting was included.
D. Data analyses	
9. Adequate description of analysed sample (inclusion and exclusion criteria)	Item was scored positively if details of the samples included in the final analysis were included (e.g. all participants, participants with complete data)
10. The analysed sample was at least medium in size	Item was scored *positively* if analysed sample was ≥250 participants

Figure S1: Forest plot depicting % energy from added sugar or sucrose and study weighting for all studies reporting on change in intake of added sugar or sucrose, together with the combined estimate.

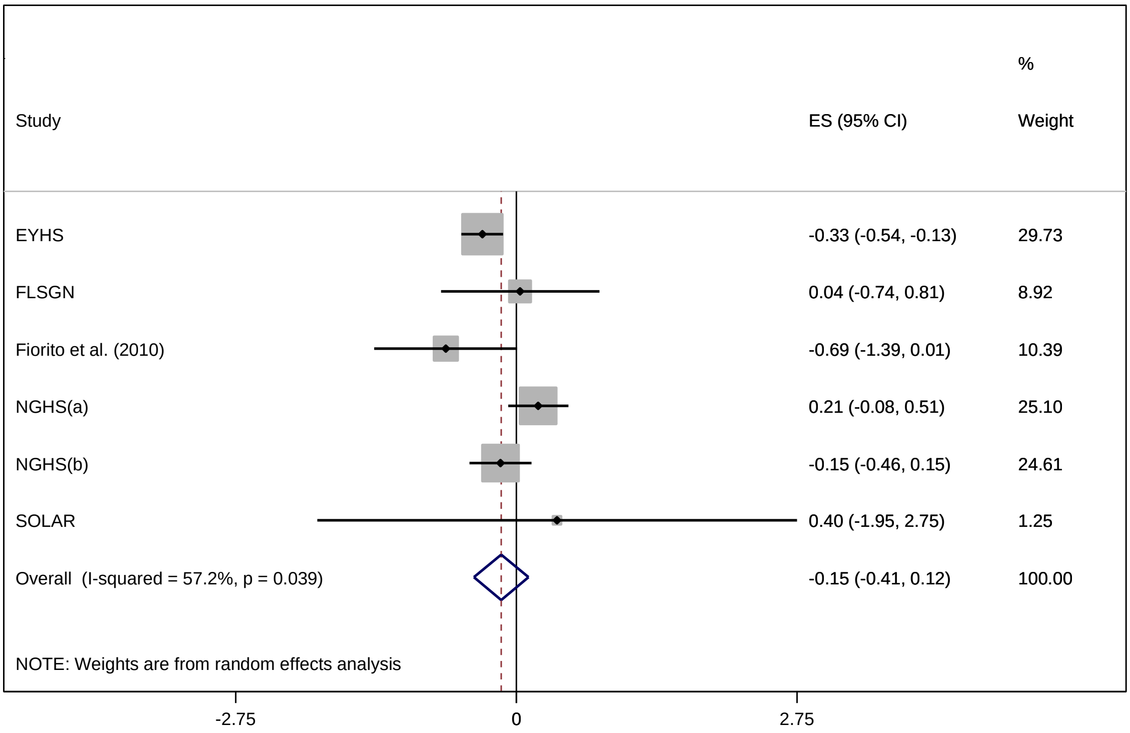

Figure S2: Forest plot depicting servings/day and study weighting for all studies reporting on change in intake of SSBs, together with the combined estimate.

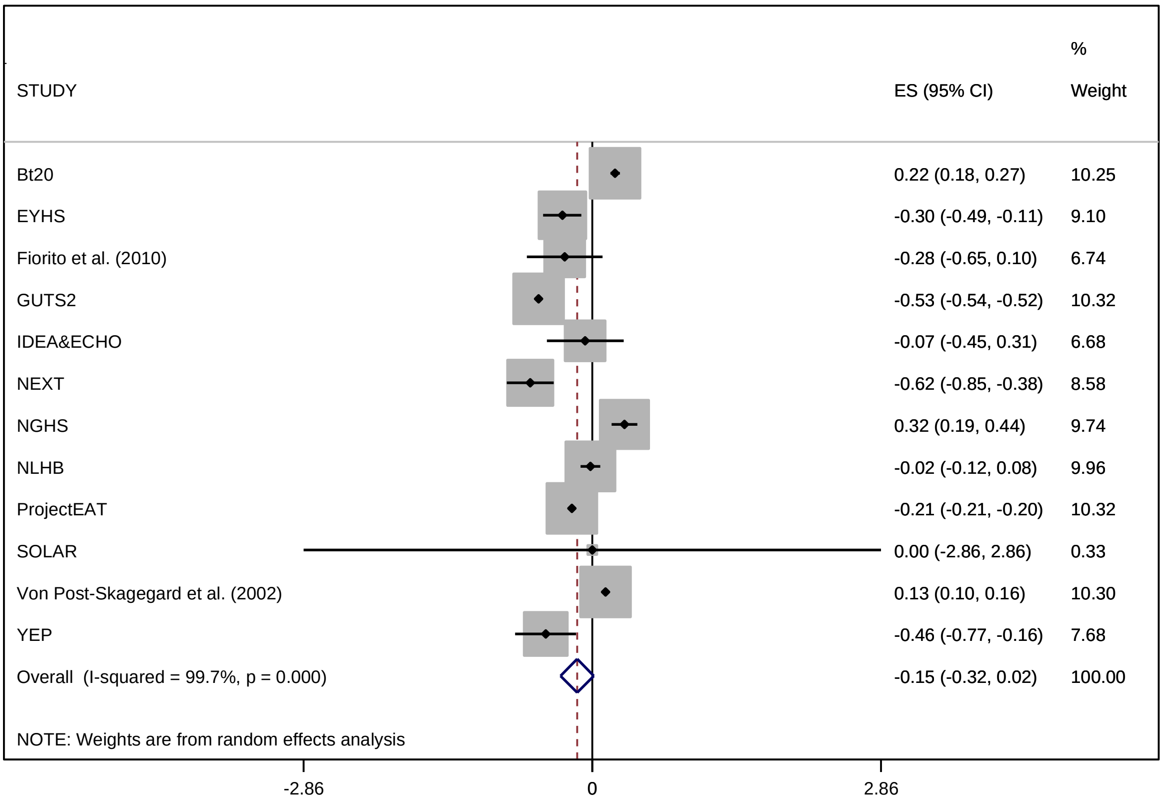

Figure S3: Forest plot depicting servings/day and study weighting for all studies reporting on change in intake of confectionery, together with the combined estimate.

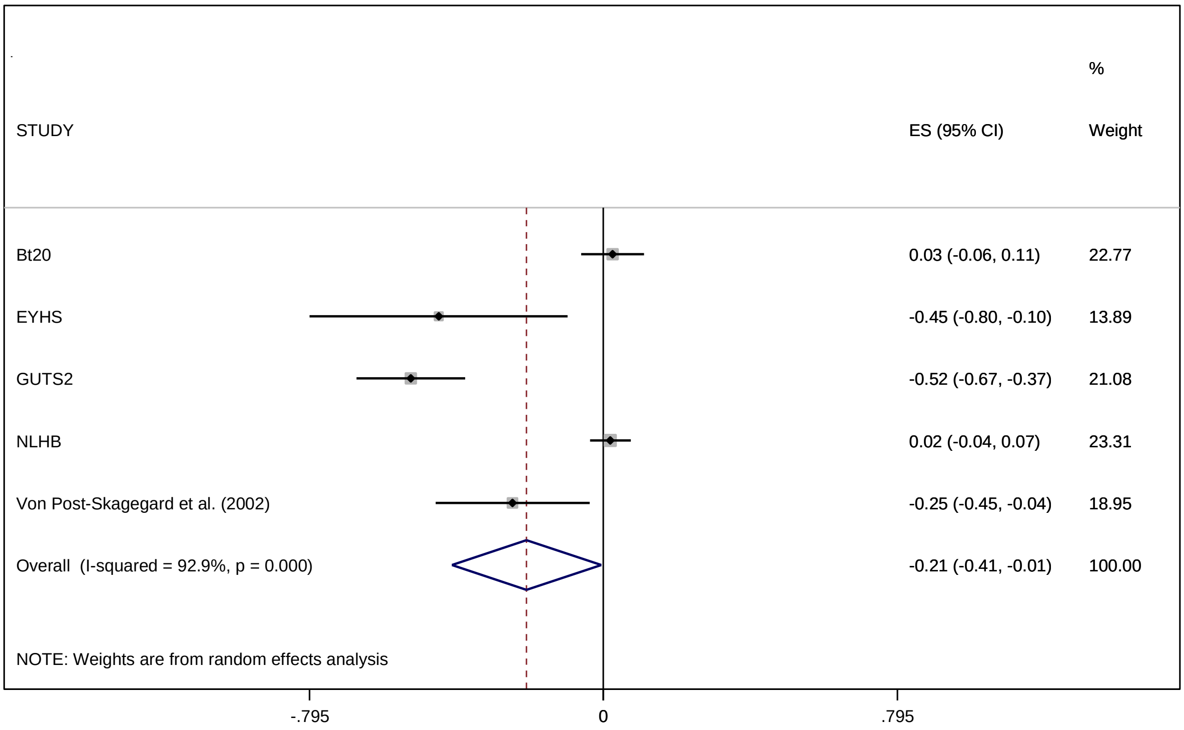

Supplement: Supplementary file 1 — Table S1: Medline search strategy Table S2: Data conversion and assumptions for each paper included in graphs and/or meta‐analysis Table S3: Quality assessment tool for longitudinal observational studies of diet Figure S1: Forest plot depicting % energy from added sugar or sucrose and study weighting for all studies reporting on change in intake of added sugar or sucrose, together with the combined estimate. Figure S2: Forest plot depicting servings/day and study weighting for all studies reporting on change in intake of SSBs, together with the combined estimate. Figure S3: Forest plot depicting servings/day and study weighting for all studies reporting on change in intake of confectionery, together with the combined estimate. [file OBR-18-1336-s001.pdf]
